# Oral Health Related Quality of Life in Diabetic Patients

**DOI:** 10.5681/joddd.2014.41

**Published:** 2014-12-03

**Authors:** Rokhsareh Sadeghi, Ferial Taleghani, Sareh Farhadi

**Affiliations:** ^1^Assistant Professor, Department of Periodontics, Faculty of Dentistry, Shahed University, Tehran, Iran; ^2^Assistant Professor, Oral & Maxillofacial Pathology Department, Islamic Azad University, Dental Branch of Tehran, Tehran, Iran

**Keywords:** Diabetes, diabetics, oral health related quality of life

## Abstract

***Background and aims.*** Diabetic patients display an increased risk of oral disorders, and oral health related quality of life (OHRQL) might affect their management and treatment modalities. The aim of the present study was to determine OHRQL and associated parameters in patients with diabetes.

***Materials and methods.*** In this study two hundred patients were recruited from the diabetes clinic in Mustafa Khomeini Hospital in Tehran, Iran. OHRQL was assessed using Oral Health Impact Profile Questionnaire (OHIP-20). Also, another questionnaire was designed which contained questions regarding participants’ knowledge about oral complications of diabetes and oral health behavior. OHRQL was categorized as low and good. Data were analyzed using logistic regression at P = 0.05.

***Results.*** Of the diabetic patients assessed, 77.5% were in good and 22.5% were in low categories of OHRQL. This quality was significantly associated with age (OR = 4.03, 95% CI = 1.63-11.29), knowledge about diabetes oral complications (OR = 18.17 95% CI = 4.42-158.6), educational level (OR = 26.31 95% CI = 4.2-1080.3), referred for dental visit by physician (OR = 3.16 95% CI = 1.48-6.69), frequency of brushing (OR = 10.29 95% CI = 3.96-31.2) and length of time diagnosed with diabetes (OR = 6.21 95% CI = 2.86-13.63).

***Conclusion.*** Oral health related quality of life was not negatively affected by diabetes mellitus in the assessed sample.

## Introduction

Diabetes is a chronic metabolic disease characterized by hyperglycemia and is caused by a defect in insulin secretion or insulin function or both.^1^ Epidemiologic studies estimate an increasing prevalence of disease from 4% in 1995 to 5.4% in 2025.^2^ In addition to many systemic adverse effects, diabetic patients encounter a high prevalence of oral complications including xerostomia, taste impairment, oral candidiasis, oral lichen planus,^3^ and periodontal disease.^4^ Especially poor or uncontrolled diabetic patients are more susceptible to periodontal disease.^5,6^ Periodontal infection may have an adverse effect on metabolic control of diabetes.^7,8^

 Considering the interaction between diabetes and oral disorders, knowledge concerning oral health related quality of life (OHRQL) in diabetic patients is an important issue. According to the World Health Organization (WHO), “quality of life is defined as an individual’s perceptions of their position in life in the context of the culture and value system where they live, and in relation to their goals, expectations, standards, and concerns.”^9^ Several studies show oral disorders may affect quality of life,^10-12^ but the effect in diabetic patients is not well-investigated. 

 Allen et al^13^ showed diabetes did not significantly affect OHRQL in the group they surveyed.^13^ Sandberg et al^14^ reached a similar result in their case-control study.^14^

 Furthermore, there is little evidence regarding awareness of the increased risk of periodontal disease among diabetic patients. Allen et al^13^ found that only 33% of diabetic patients were aware of their increased risk for periodontal disease. Sandberg et al^14^ reported the awareness among 17% of their study population. 

 Studies of diabetic patients’ well-being and oral health related complications might affect their management and finally improve their satisfaction with life. Therefore, the present study was designed to determine OHRQL and its associated factors in diabetic patients. 

## Materials and Methods

 The participants of this analytical cross-sectional study were recruited from patients attending the diabetes clinic in Mustafa Khomeini Hospital, Tehran, Iran, by convenience sampling during the year 2011. Two-hundred well-controlled diabetic patients (HbA_1_c < 8) were included in this survey.^1^ The selected participants had a confirmed diagnosis of diabetes and were under treatment with frequent recall visits. Written informed consents to participate in the study were obtained from all of the participants. 

 General information of the participants including age, gender, educational level, type of diabetes, length of time diagnosed with diabetes and smoking habit were recorded. Questions related to oral health care such as frequency of brushing, frequency of dental visits, and being referred to a dentist by their physician were answered by the participants. 

 Standard oral health impact profile (OHIP-20) questionnaire, which is the shortened version of OHIP, was used for the evaluation of OHRQL.^15,16^ The questionnaire consisted of 20 multiple-choice questions assessing oral-health related problems in seven conceptual domains including functional limitation, pain, physiological discomfort, physical disability, psychological disability, social disability, and handicap. Based on the presence/absence of the problem and its severity, the answers were classified into 5 groups and each answer took the score of 1 to 5 as: never (the problem was never experienced) = 5, seldom = 4, occasionally = 3, most of the times = 2, and always (the problem always existed) = 1. The total score was calculated for each individual summing the scores for each question, which could vary from 20 to 100 (The highest and the lowest possible scores were 100 and 20, respectively). A total score of 100 indicated absence of difficulty or problem, whereas lower scores suggested that the respondent experienced some degree of oral-health related problems. OHRQL was categorized into two levels as low for scores of 20-59, and good for 60-100. 

 In addition to OHIP-20, another questionnaire related to participants’ knowledge on oral complications of diabetes was administered to the participants, which included descriptive and multiple choice questions. The validity of this questionnaire and answers to its questions were assessed by the Delphi method, i.e. eight expert periodontists reached an agreement on all items and gave them a score. Both questionnaires were in Persian. 

### Statistical Analysis

 Data were summarized using frequency (%) for qualitative variables and mean (SD) for quantitative variables. Logistic regression analysis was used to assess the association between OHRQL and the variables. The level of significance was set as p < 0.05. All analyses were performed using SPSS version 15 (SPSS Inc., Chicago, USA). 

## Results

 200 diabetic patients, consisting of 88 men and 112 women with a mean age of 55.2 years, participated in this survey. The frequencies of demographic variables assessed in the studied sample are given in Table 1. 

**Table 1:** Socio-demographic data and dental history of participants

variable	classification	frequency	percentage
age	50 years old and less	73	36.5%
	More than 50 years old	127	63.5%
sex	Male	88	44%
	female	112	56%
Educational level	No academic	141	70.5%
	academic	59	29.5%
Smoking status	Smoker	101	50.5%
	Non smoker	99	49.5%
Knowledge	Good	73	36.5%
	low	127	63.5%
Type of diabetes	1	12	6%
	2	160	80%
Duration of disease	Lower than 10 years	136	68%
	10 years and more	64	32%
Cause of tooth extraction	Mobility	4	2%
	caries	54	27%
	Mobility and caries	89	44.5%
	No missing tooth	53	26.5%
Frequency of brushing	No brushing	99	49.5%
	Once a day	87	43.5%
	Twice a day	14	7%
Referring for dental visit by physician	Yes	142	71%
	No	58	29%
Frequency of dental visits	G1^§^	166	83%
	G2^§^	34	17%
^§^individuals who have regular dental check up			
^§§^Individuals who have not regular dental check?up and visit the dentist only when have a dental problem			

 With regard to satisfaction with life, the results indicated 155 patients (77.5%) had good OHRQL (95% Confidence Interval [CI] = 71.7%-83.3%) and 45 patients (22.5%) had low OHRQL (95% CI= 16.7%-28.3). 

 No significant association was found between OHRQL and gender, smoking habit, the type of diabetes, and the frequency of dental visits. However, OHRQL was significantly related to age, knowledge of the link between diabetes and oral complications, educational level, being referred for dental visits by their physicians, frequency of brushing, and the length of time diagnosed with diabetes (P < 0.05 in all cases; Table 2). 

 According to odds ratios (OR), those diabetic patients who had poor knowledge of oral manifestations of diabetes (OR = 18.17, 95% CI = 4.42-158.6), lower educational level (OR = 26.31, 95% CI = 4.2-1080.3), and poor oral hygiene (OR = 10.29, 95% CI = 3.96-31.2) were far more likely to have low OHRQL. Also those who were 50 years of age and older (OR = 4.03, 95% CI = 1.63-11.29), diagnosed with diabetes for more than 10 years (OR = 6.21, 95% CI = 2.86-13.63), and not referred to dentists by their physician (OR = 3.16, 95% CI = 1.48-6.69) were also more likely to have low OHRQL (Table 2). 

**Table 2:** The correlation between OHRQL and variables.

		OHRQL		OR	P value
Variables		Good	Low	95%CI	
		N = 155	n = 45		
Gender	Female	82(52.9%)	30(66.7%)	1.78 (0.85 to 3.85)	0.102
	Male	73(47.1%)	15(33.3%		
Age	50 years old and less	66(42.6%)	7(15.6%)	4.03 (1.63 to 11.29)	<0.001
	More than 50	89(57.4%)	38(84.4%)		
Knowledge	Good	71(45.8%)	2(4.4%)	18.17 (4.42 to 158.6)	<0. 001
	Low	84(54.2%)	43(95.6%)		
Educational level	No academic	97(62.5%)	44(97.7%)	26.31 (4.20 to 1080.3)	<0. 001
	academic	58(37.5%)	1(2.3%)		
Type of diabetes	1	9(5.8%)	3(6.7%)	1.28 (0.21 to 5.52) a	0.719
	2	127(81.9%)	33(73.3%)		
Smoking status	Smoker	74(47.7%)	27(60%)	1.64 (0.80 to 3.44)	0.148
	Non Smoker	81(52.3%)	18(40%)		
Being referred for dental visit	yes	119(76.7%)	23(51.1%)	3.16 (1.48 to 6.69)	<0. 001
	no	36(23.3%)	22(48.9%)		
Frequency of brushing	Once and more	95(61.3)	6(13.3%)	10.29 (3.96 to 31.2)	<0. 001
	No brushing	60(38.7%)	39(86.7%)		
Frequency of dental visits	G1^§^	126(81.3%)	40(88.8%)	1.84 (0.64 to 6.48)	0.232
	G2^§§^	29(18.7%)	5(11.2%)		
Duration of disease	<10 years	120(77.4%)	16(35.6%)	6.21 (2.86 to 13.63)	<0.001
	≥10 years	35(22.6%)	29(64.4%)		
^§^individuals who have regular dental check-up					
^§§^Individuals who have not regular dental check-up and visit the dentist only when have a dental problem					
a: Type 2 versus Type1 diabetes					

## Discussion

 There have been many studies on the quality of life among diabetic patients but few have evaluated OHRQL. In the current survey, the OHIP-20 questionnaire was employed to evaluate OHRQL. The results indicated OHRQL among most diabetic patients (77.5%) as being in the “good” category. Therefore, it seems that oral complications of diabetes have not adversely affected OHRQL in these patients. This is in line with the study of Allen et al^13^ on 101 diabetic patients. In addition, Sandberg et al^14^ in their case-control study on 204 Swedish diabetic and non-diabetic patients using SF-36 questionnaire (Short Form General Health Survey Measure; Ware-Steward) demonstrated that patients in both study groups were satisfied with their oral health. Furthermore, in another study with the same design and sample size, Sandberg and Wikblad^12^ reported that although general health related quality of life (HRQL) was similar in both study groups, the non-diabetic individuals demonstrated a better health status in regards to HRQL scores compared with the diabetic group. Certain oral factors e.g. xerostomia played a negative role in the low HRQL status. 

 The results of the present study showed that sex, type of diabetes, smoking and frequency of dental visits had no significant effect on OHRQL; variables including age, level of education, frequency of brushing, duration of diabetes, being referred to a dentist by a physician had some effects on scores of OHRQL in our study. There are few studies related to the effect of these factors on OHRQL; however, some research on general quality of life showed conflicting results in this regard. According to a review by Wandell,^17^ age, sex, ethnicity, socioeconomic factors, educational level, and income have been reported to have no specific effects on diabetic HRQL. However, the results of a study on 699 diabetic patients demonstrated a higher quality of life in younger aged, male gender and more highly educated patients.^18^ Also, according to another research on 1070 type 1 diabetic patients, HRQL decreased with increasing age.^19^

 Patients who brushed once a day or more had better OHRQL in comparison to patients with less frequent brushing in the present study, which is in line with findings of Sandberg et al.^14^ Also, those who were referred to a dentist by their physicians experienced good OHRQL. These findings can reflect the effect of oral health care and dental treatments on the improvement of OHRQL in diabetic patients. 51.5% of subjects in our study reported a brushing frequency of once or twice a day. However, in the survey of Sandberg et al,^14^ more than 90% of cases had daily brushing habits. In the study on Finnish diabetics by Karikoski et al,^20^ 38% of 258 subjects reported brushing more than once a day, 44% brushing once a day and 17% less than once a day. 

 Referring diabetic patients to dentists through their physicians is another parameter considered in the present study. 71% of our patients were referred, which is higher than 16% reported by Karikoski et al.^20^

 In addition, there was a relationship between the awareness of oral complications of diabetes among patients and their OHRQL. From 155 diabetic patients with good OHRQL, 45.8% were in good classification of knowledge on the issue. However, out of 45 patients with low OHRQL only 4.4% were classified in the knowledge group of good. 

 Although this study showed high oral health related quality of life in diabetic patients, limitations should be considered in the interpretation of the results. Firstly, the patients were not clinically examined and the results were exclusively based on the information provided by the questionnaires. Secondly, these results were collected from patients who were referred to one hospital only, so it cannot be extended to all diabetic patients. In addition, various biases may affect the results of surveys based on questionnaires. For example, it seems that patients usually reply to questions with answers which seem more reasonable. 

 Based on the results of this survey, it seems that, OHRQL is not adversely affected by the presence of diabetes mellitus; however, there might be a relation between some variables and oral health related quality of life. It seems that dentists and physicians play an important role in improving diabetic patients’ knowledge regarding oral complications and their effect on their quality of life. Also, it is recommended that referring to a dentist could be a part of diabetes treatment protocol. 

## Acknowledgement

 This work is part of a thesis for the degree of DDS. The authors would like to thank Dr. Z. Tamimi, Dr. Z. Sabet, Mr. N. Valaee and Dr. Asghari Jafar-Abadi for their helpful assistance. The authors declare that they have no conflict of interests. 
